# Understanding Motivating Factors for COVID-19 Vaccination in Families Defaulting from Childhood Immunization: A Mixed-Methods Study in Pakistan

**DOI:** 10.3390/pediatric18010024

**Published:** 2026-02-05

**Authors:** Kifayat Ullah, Chukwuma Mbaeyi, Javeria Saleem, Muhammad Ishaq, Muhammad Rana Safdar, Aslam Pervaiz, Tamkeen Ghafoor, Mumtaz Ali Laghari, Sumbal Hameed, Fatima Majeed, Usman Javed Iqbal, Amjad Mehmood

**Affiliations:** 1 Federal Directorate of Immunization, Islamabad 44000, Pakistan; drsafdar64@yahoo.com; 2 Integral Global Health, Islamabad 44000, Pakistan; aslam_pervaiz92@yahoo.com (A.P.); tamkeen@integralglobal.net (T.G.); drmumtazalilaghari@gmail.com (M.A.L.); amjadmehmoodahd@gmail.com (A.M.); 3 Institute of Social and Cultural Studies (ISCS), University of the Punjab, Lahore 54590, Pakistan; fatimamajeed2@gmail.com; 4 Polio Eradication Branch, Global Immunization Division, US Centers for Disease Control and Prevention, Atlanta, GA 30329, USA; 5 Department of Public Health, Green International University, Lahore 55150, Pakistan; javeria.hasan@hotmail.com; 6 QA Higher Education Business School-Ulster University Partnership Program, Birmingham Campus, Birmingham B3 3PL, UK; m.ishaq@ulster.ac.uk; 7 National Emergency Operations Center (NEOC), Islamabad 44000, Pakistan; sumbalhameed5682@gmail.com; 8 National Stop Transmission of Polio, Islamabad 44000, Pakistan; 9 Postgraduate Medical Institute, Gulab Devi Teaching Hospital Lahore, Lahore 54600, Pakistan; sh.usmanjavediqbal@gmail.com

**Keywords:** COVID-19, childhood vaccination, defaulter children, partially immunized children, routine immunization

## Abstract

Background: The COVID-19 pandemic significantly disrupted health systems, including the delivery of childhood immunizations. This study assessed COVID-19 vaccination coverage in families of children with incomplete routine immunization and explored why adults accepted COVID-19 vaccines despite skipping routine vaccination for their children in the district of Swat, Khyber Pakhtunkhwa, Pakistan. Methods: A mixed-methods study was conducted in Swat District during March 2022–April 2023. A cross-sectional survey assessed COVID-19 vaccination in household members of children under 2 years of age for whom vaccination registries showed missed routine vaccinations. In-depth interviews with 18 household members explored motivations for vaccine acceptance through thematic analysis. Results: Among 249 families of children with incomplete immunization found through vaccination records, 237 families (88% response) were interviewed. Among 382 children below 2 years of age in these families, 29.5% (n = 113) were fully vaccinated, 67.5% (n = 258) were incompletely vaccinated according to age, and 2.9% (n = 11) had not received any vaccine. Data from 237 of the defaulter children—one per household—was included in further analysis. Among household members above 12 years of age, 87% (964/1103) of males and 82% (n = 901/1093) of females were vaccinated against COVID-19. Households with at least one fully vaccinated child were significantly more likely to have vaccinated family members. Multivariable analysis showed maternal COVID-19 vaccination (AOR 2.08, 95% CI: 1.15–3.76) and urban residence (AOR 2.08, 95% CI: 1.14–3.79) were associated with full childhood vaccination. In-depth interviews revealed that key motivators for COVID-19 vaccination included the perception that it was mandatory, house-to-house vaccination, and fear of hospitalization or death. Conclusions: Vaccine requirements and ease of access to vaccination services enhanced coverage with COVID-19 vaccines among families of children with incomplete routine immunization. Ethical use of vaccine requirements and community education to enhance levels of risk perception of vaccine-preventable diseases could potentially improve childhood immunization.

## 1. Introduction

The COVID-19 pandemic has had a major global impact, resulting in millions of deaths directly or indirectly [1]. Disruption of routine health and immunization services also adversely affected the control of vaccine-preventable diseases (VPDs) in both wealthy and less affluent nations [2], especially in countries where the numbers of unvaccinated or partially vaccinated children were already high. The term “partially vaccinated” or “defaulter child” refers to a child who has initiated the routine vaccination series but has not completed the recommended vaccination schedule due to any reason [3,4].

Although considerable progress has been made globally in vaccinating children under two years of age, developing countries face numerous challenges in access and utilization of vaccines offered through the Expanded Programme on Immunization (EPI). Many children in impoverished countries do not receive even a single dose of the recommended vaccination schedule, and some receive the initial doses but do not complete the vaccination series [5]. Completion of the recommended vaccination schedule is of paramount importance for developing adequate immunity against vaccine-preventable diseases. Failure to complete the schedule leaves individuals at risk of contracting the disease prevented by the vaccine and may provide a false sense of protection that could influence their risk perception [6]. According to the World Health Organization (WHO), in 2023, 14.5 million eligible children worldwide did not receive the initial dose of the DPT (Diphtheria, Pertussis, and Tetanus) vaccine and an additional 6.5 million received partial doses [7]. The gaps in vaccination coverage are especially high in low-income regions such as sub-Saharan Africa, having coverage less than global targets and high number of zero-dose children. The occurrence of numerous outbreaks of VPDs in developing countries and continued transmission of poliovirus in Pakistan and Afghanistan underscores the presence of a significant number of unvaccinated (zero-dose) and partially vaccinated (defaulter) children in these countries. In Pakistan alone, nearly 0.4 million zero-dose children were reported in 2023 [8].

Coverage for DPT-3 usually varies widely by country and WHO region, and the COVID-19 pandemic adversely impacted all regions except for the Western Pacific region. During 2019–2020, coverage in the African region was only 72% compared with 95% in the Western Pacific region. Although the global dropout rate from DPT-1 to DPT-3 remained stable at 4–5% during 2019–2020, the number of zero-dose children increased in most regions. The African region saw an increase from 7.1 million to 7.7 million, the Americas from 1.6 million to 1.7 million, the Eastern Mediterranean region from 1.8 million to 2.3 million, the Southeastern Asian region from 2 million to 4.1 million, and the Western Pacific region from 0.9 million to one million. Only in the European region did the number remain stable at approximately 300,000 children [9].

Pakistan has made progress in its vaccination indicators but significant gaps in quality remain. Data from a 2017–2018 demographic survey indicated that 66% of children aged 12–23 months had received full immunization coverage, which increased to 76% in 2021 and remained the same in 2023 but still fell below recommended WHO standards [10,11]. BCG coverage at birth stood at 88% nationwide, while measles vaccine coverage was 73%, indicating a dropout rate of 17% between BCG and measles vaccination. This drop-out rate represents children who have visited vaccination centers at least once and received one or more vaccine doses but failed to complete the vaccination series [12]. Globally, multiple complex factors contribute to incomplete vaccination among children, including the healthcare system’s inability to consistently provide vaccines and vaccination services, staff absenteeism and shortage of clinic personnel (70%), limited operational hours at vaccination facilities (56%), and the challenges of accessing distant locations (15%) [13]. Various program policies including appointments, specific vaccination days, limits on per day vaccination, and long waiting times also have a direct impact on vaccine coverage and utilization. A study conducted in 2020 by Butt M et al. found that approximately 12.6% of children in Pakistan were unvaccinated due to the considerable distance to EPI centers and 30% of women perceived accessing healthcare facilities as challenging [14].

Despite the obstacles to achieving high levels of childhood vaccination coverage, Pakistan and other low and middle-income countries (LMIC) achieved high coverage with COVID-19 vaccination among their adult populations. Pakistan developed a rapid strategy for COVID-19 vaccination led by the National Command and Operational Center (NCOC) and supported by involvement of health staff specifically from the polio program and EPI. Strong political commitment and extensive risk communication campaigns also played important roles. In Pakistan, during the initial days of COVID-19 vaccine availability, there was a high level of community eagerness for receiving the vaccine as evidenced by the high numbers of individuals vaccinated [15]. Considering the contrast in this scenario to attitudes towards childhood routine immunization, we decided to investigate why the acceptance of COVID-19 vaccines appeared to be significantly higher than that of childhood vaccines against other VPDs.

We conducted this study to assess the COVID-19 vaccination status of families with at least one child under two years of age who had incomplete routine immunization and to explore the factors influencing their decision to vaccinate adult family members against COVID-19 while neglecting to complete their children’s routine immunization. The characteristics of children with incomplete vaccination and the reasons for non-vaccination are published in a separate manuscript [16].

## 2. Methods

### 2.1. Study Design

A mixed-methods study was conducted in Swat District of Khyber Pakhtunkhwa, Pakistan, from March 2022 to April 2023. The study had two components conducted simultaneously. Initially, a cross-sectional coverage survey was conducted to determine the COVID-19 vaccination status of the families of children who had not completed the recommended EPI vaccination schedule. This quantitative component aimed to ascertain whether families refraining from vaccinating their children against VPDs were also abstaining from vaccinating adults against COVID-19 and vice versa. In the second component, a qualitative phenomenological study was conducted to explore the motivating factors that led families to vaccinate adults against COVID-19 while choosing not to vaccinate their children with routine vaccines provided through the EPI system.

### 2.2. Study Population

For the cross-sectional coverage survey, the study population comprised families of children under the age of two years who had not completed the EPI recommended schedule for their age, including all the household members eligible for COVID-19 vaccination. In Pakistan, vaccines are administered against twelve VPDs. These include Bacillus Calmette–Guérin (BCG), Oral Polio Vaccine (OPV), Hepatitis B vaccine, Rotavirus vaccine, Pneumococcal Conjugate Vaccine (PCV), Pentavalent vaccine (Diphtheria, Tetanus, Pertussis, Hepatitis B, Hemophilus influenzae type b (Hib)), Inactivated Polio Vaccine (IPV), Typhoid Conjugate Vaccine (TCV), and Measles-Rubella vaccine (MR). For this study, a “defaulter” was defined as any child aged 1 to 23 months who had initiated but missed one or more recommended routine immunization doses. At the time of the study, COVID-19 vaccination was approved for individuals aged 12 years and above; therefore, all household members of age 12 years and above living with identified defaulter children were considered “eligible” family members.

### 2.3. Sample Selection

Stratified random sampling was employed to select union councils (UCs), which are administrative subdistricts units, within the district of Swat. Initially, the 65 UCs of Swat were categorized into two groups: urban and rural. Subsequently, four UCs (two from each category) were chosen using a simple random sampling technique. Within the selected UCs, children who had defaulted on their vaccination schedules were identified using records from the EPI and Lady Health Worker’s (LHW) registers. For every identified defaulter child, an interview team visited the households and invited caregivers to participate in the study through oral consent. When the caregiver consented to participate, the team conducted a survey among the household members targeting all children younger than 2 years of age and all individual members older than 12 years.

In addition, all caregivers older than 18 years of age, residing permanently in the same household and having blood relation with the child and who were willing to interview, were invited to participate in semi-structured in-depth interviews to assess motivating factors for COVID vaccination. A total of 18 semi-structured interviews were conducted, and further interviews were discontinued upon saturation of data, i.e., as participants began to share the same information already expressed in previous interviews.

### 2.4. Data Collection

For the coverage cross-sectional survey conducted, the team used a structured questionnaire that included information about the vaccination status of the enrolled defaulter child (zero dose, defaulter, or fully vaccinated), EPI vaccination status of other children under two years of age in the household, and the number of COVID-19 vaccine doses received by all individuals above 12 years of age in the household. As part of the survey, other information collected included gender and relationship of the interviewee with the defaulter child, family type (joint or nuclear), as well as demographic details of the defaulter child such as age, gender and birth order. Information on routine vaccination was based upon vaccination registers of the respective vaccination centers, child’s vaccination card and parental recall (for children with zero doses). The source of COVID-19 vaccination status was individual’s recall supported by information from other family members. Data was collected by the researchers through door-to-door visits. Information on defaulter children was obtained from the relevant vaccination centers, after which their households were visited to collect the required data. A structured data collection form was used, and all information was recorded in writing at the time of the visit. For the in-depth interviews, a semi-structured interview guide was employed to ensure comprehensive coverage of relevant topics. To ensure no valuable information was left out, the interviews were recorded and detailed field notes were taken.

### 2.5. Ethical Approval

The study was conducted following ethical review and approval by the Institutional Ethics Review Board at the University of Punjab, Pakistan, on 27 March 2023, with reference number D/46/FIMS. Verbal informed consent was obtained from all study participants.

### 2.6. Data Analysis

Data was analyzed with SPSS version 26.0. Descriptive statistics such as frequencies and percentages were computed to describe the demographic variables of the participants and the vaccination status of children and family members. Bivariate analyses, including Chi-square tests, were conducted to determine the associations between routine childhood immunization status and categorical variables, such as age group, sex, family type, and the COVID-19 vaccination status of family members. Independent variables with *p*-values < 0.05 in Chi-square analysis were included in a binary logistic regression model to determine factors that were independently associated with having one or more fully vaccinated children.

Subsequently, the covariates included in the multivariate model were child’s age, family type (joint or nuclear), and male family members’ COVID-19 vaccination status. Odds ratios and then adjusted odds ratios (AORs) with 95% confidence intervals were calculated. The McNemar’s test was also applied to determine the within-household concordance of the COVID-19 vaccination status of male and female family members. A *p*-value of <0.05 was taken as statistically significant.

**Figure 1:**
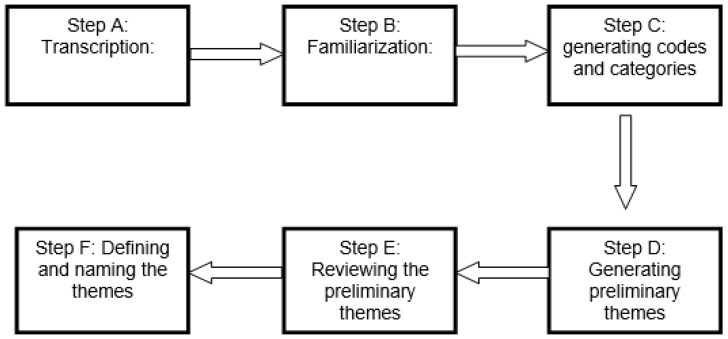
Flow chart of qualitative data analysis.

Qualitative data from the second component of the study was subjected to thematic analysis. The principal investigator conducted the initial coding and developed preliminary themes. To enhance consistency and accuracy, a co-author independently reviewed a subset of the coded data and themes, and any discrepancies were resolved through discussion and consensus. The analytic process commenced with the transcription of all 18 recorded interviews and the incorporation of field notes into the dataset. Familiarization with the data was achieved through repeated listening and data review, leading to identification of initial codes reflecting relevant themes. These codes were subsequently organized and grouped into categories. Final themes were then identified and named to elucidate their essence. The themes were presented using self-explanatory, easily understandable labels, ensuring clarity and fidelity to the core concepts. Excerpts and quotes from the interviews were included to illustrate and support the findings (Figure 1). To facilitate reference and contextual understanding while maintaining participants’ confidentiality, the demographic attributes of each participant quoted within the study were added to their serial number. For instance, Participant–01, 35, M refers to Participant Number 1, a male with an age of 35 years, and Participant–4, 60, F indicates Participant Number 4, a female aged 60 years.

## 3. Results

### 3.1. Identification of Participants

The review of childhood immunization records in the four UCs (Saidu Sharif, Qambar, Kokarai, and Islampur), randomly selected, identified 269 defaulter children below 2 years of age. All the families of these children were approached for participation in the study; 32 families (12%) refused to participate or were not available, resulting in 237 families surveyed agreeing to participate in the study, a response rate of 88%.

**Figure 2:**
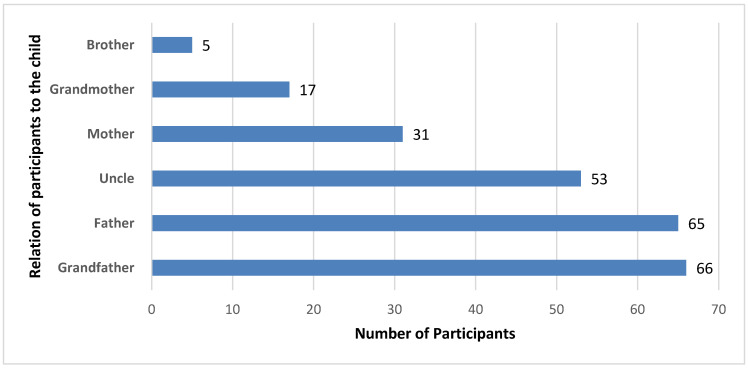
Relationship of the participants with the defaulter child (n = 237).

During the vaccination assessment, 382 children below 2 years of age were identified in the 237 survey-respondent families. Among these children, 29.5% (n = 113) were fully vaccinated, 67.5% (n = 258) were incompletely vaccinated according to age, and 2.9% (n = 11) had not received any EPI vaccine dose. Demographic data from 237 defaulter children—one per household—was used for analysis of factors that could have influenced COVID-19 vaccination in their family members. The information was collected from various family members, most of them were either grandfather or father (Figure 2).

### 3.2. Characteristics of Defaulter Children (Children Partially Vaccinated)

Among the 237 defaulter children assessed, 124 (52%) were male and 113 (48%) were female. Their mean age was 10.5 months (range: 3–24 months). Most children were aged between 6 and 12 months (59.1%), followed by those aged 12–18 months (20.7%). A smaller proportion were under 6 months (11.8%) or over 18 months (8.4%). Of the total, 53.6% resided in urban UCs, while 46.4% were from rural UCs and most of the households had joint family setups (87.8%). Analysis of birth order indicated that most defaulter children were second-born (33.7%) or first-born (21.5%), followed by third-born (15.6%), fourth-born (11.3%), fifth-born (6.7%), sixth-born (6.3%), and seventh-born (4.6%). Approximately 58.6% of households had only one child under the age of two years, while 28.7% had two such children, and 12.7% had three or more.

For adult vaccination status with COVID-19, 54.4% of the families reported that all male members were fully vaccinated (two or more doses), and 67.5% reported that all female members were fully vaccinated (Table 1).

We observed several significant relationships between routine immunization status in children under two years of age and demographic and household factors (Table 2). A significant correlation was found between the child’s age and complete vaccination (*p* = 0.047). Higher proportions of children between 6 and 12 months were age appropriately vaccinated (62.7%), compared with children over 12 months (11% for 12–18 months and 12% for >12 months) (Table 2).

Household size was significantly associated with immunization completion (*p* < 0.001). Families with only one child below 2 years were significantly more likely to have no fully vaccinated child, while families with two or more children had a substantially higher proportion of complete vaccination (Table 2). We did not observe a significant relationship with routine immunization status (*p* = 0.729) and child’s gender or urban versus rural settings (*p* = 0.739).

An overall relationship was observed between adult males who had received at least two doses of the COVID-19 vaccine and the routine vaccination status of children (*p* = 0.022). Households where all the male members had received two or more doses of the COVID-19 vaccine had a higher chance of their children being fully immunized, suggesting a possible association between general health-seeking behavior and adherence to vaccination. However, no significant association was noted for the COVID-19 vaccine status of female members (*p* = 0.684) (Table 2).

We included child’s age, family types (joint or nuclear), and male family members with completed COVID-19 vaccination in binary and multivariate regression analysis. As shown on Table 3, age of child, family type, and male members’ COVID-19 vaccination status remained significant. After adjusting for other factors, children aged 13 to 18 months were not significantly less likely to be fully vaccinated than those aged less than six months (AOR = 0.338, *p* = 0.052). Although this finding was not statistically significant, it suggests a potential trend toward delayed or incomplete vaccination with increasing age. With regard to family composition, nuclear families were significantly less likely to have well-immunized children compared with joint families (AOR = 0.361, *p* = 0.014). COVID-19 vaccination status in male family members did not show a significant association. Families in which all male members had not received two or more COVID-19 vaccine doses completely were not significantly less likely to have children who were fully vaccinated (AOR = 0.576, *p* = 0.065).

### 3.3. COVID-19 Vaccination Status Among Eligible Family Members

**Figure 3:**
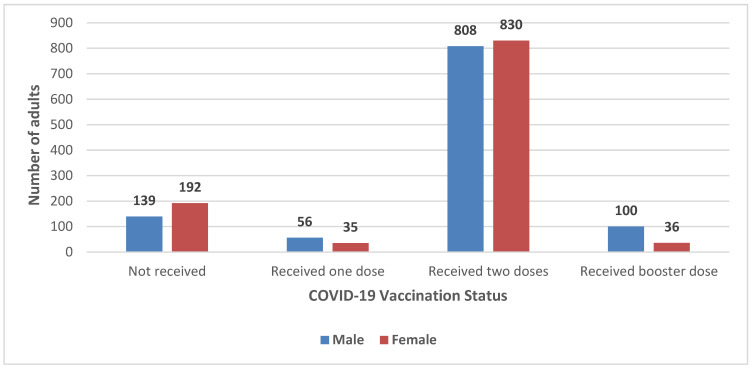
COVID-19 vaccination status of family members of the defaulter child (male n = 1103, Female n = 1093).

Within the 237 families with defaulter children assessed in this study, 2196 individuals were eligible for COVID-19 vaccination (i.e., above 12 years of age), 1103 (50.2%) males and 1093 (49.7%) females. Of these, 87% of males (n = 964) reported having received COVID-19 vaccine compared with 82% (n = 901) of females (*p* = 0.001). Among vaccinated males, 5% (n = 56) had received only one dose, 73% (n = 808) two doses, and 9% (n = 100) had received a booster dose. Among eligible females, 3% (n = 35) received only one dose, 76% (n = 830) received two doses, and 3% (n = 36) also received a booster dose of COVID-19 vaccines (Figure 3).

Within the same household, there was discordance between the COVID-19 vaccination status of male and female family members (*p* =< 0.01, McNemar’s test). Specifically, although 116 families reported that all male and female members had received two or more doses of the COVID-19 vaccine, 64 families had vaccinated all females but not males, and 13 families had vaccinated all males but not females.

### 3.4. Qualitative Analysis of In-Depth Interviews

The in-depth interviews were conducted primarily with male caregivers; fathers accounted for 27.4% of respondents, followed by grandfathers (27.8%), uncles (22.3%), mothers (13.08%), grandmothers (7.1%), and brothers (2.1%). The mean age of the respondents was 43 years (range: 18–75 years).

The following major themes were identified from the in-depth interviews.

### 3.5. Mandatory COVID-19 Vaccination

The theme of “mandatory COVID-19 vaccination” highlights that COVID-19 vaccination efforts in the study area involved a sense of compulsion or pressure on adults to get vaccinated. It appears that the interviewees perceived COVID-19 vaccination as mandatory and indicated that vaccination teams were actively inquiring about their vaccination status.

“*My wife is ill and has chronic diseases, we have to travel to a nearby city for her treatment and everywhere the teams are asking for COVID-19 vaccination status. Therefore, we did vaccination on time to avoid these hurdles during travel and in the hospital*”.

(PRC–02,55, M)

The above statement highlights a common reason for adults to get vaccinated against COVID-19: the need to access various services, including healthcare, that could have required proof of vaccination. The participant’s description of how COVID-19 vaccination teams were actively confirming people’s vaccination status at airports, bus terminals, hospitals, government offices, and even at roadside check-posts highlights the comprehensive efforts undertaken to encourage and facilitate vaccination.

“*The government has imposed vaccination against COVID-19 on us, and no one can fight against the government, and we are forced to be vaccinated*”.

(PRC–13,75, M)

The implementation of mandatory COVID-19 vaccination for school and university admissions was another measure taken by the government to ensure a higher vaccination rate in the population.

### 3.6. Ease of Access to Vaccination Against COVID-19

The ease of getting vaccinated against COVID-19 at home through the efforts of Lady Health Workers and vaccination teams was identified as a significant facilitator in achieving higher vaccination rates. This approach brought the vaccine directly to people’s doorsteps, making it more convenient for individuals and families to access vaccination services. It not only simplified the process but also ensured follow-up for the second dose, which was crucial for vaccine effectiveness.

The contrast between the need to visit a vaccination center for childhood vaccination and the convenience of receiving COVID-19 vaccines at home highlighted a significant difference in accessibility and approach. For childhood vaccination, families need to visit healthcare facilities, which can be challenging for various reasons, including work schedules, competing commitments, access to transportation, and other responsibilities. On the other hand, the proactive approach of healthcare workers visiting homes to administer COVID-19 vaccines was found to be more effective in reaching and vaccinating family members.

“*For completing the vaccination schedule of the child, we have to visit the vaccination center located at the health facility. For this, we have to manage proper timing while for vaccination against COVID-19, none of our family members visited the vaccination center. We all received vaccines here at our home*”.

(PRC–08, 30, M)

Multiple campaigns were planned for COVID-19 vaccination activities. Mobile teams were deployed in the community to visit houses door–to–door to vaccinate every eligible household member against COVID-19. The teams were remunerated for the activity and proper monitoring and supervision plans were implemented. Teams were directed to prepare comprehensive microplans before campaigns. All team members were trained before campaigns on vaccination techniques, vaccine management, cold chain storage, and interpersonal communication to persuade community members to accept vaccination against COVID-19. If any refusals to vaccination were found, teams tried to persuade the individuals, but if they were still unsuccessful in doing so, then they reported such households to their supervisors for a follow-up visit.

“*When the vaccination team visited our home, they vaccinated all the members of the family but my father refused to be vaccinated against COVID-19, the team tried to convince him but in vain even I tried but he refused, later on the doctor (Supervisor) posted at nearby health facility visited our home and convinced my father for vaccination against COVID-19*”.

(PRC–15, 52, M)

The campaign was properly monitored and administrative agencies other than the health department, such as police and district administration, were also engaged in the campaign to make sure that every home was visited by the mobile vaccination teams.

### 3.7. Fear of Hospitalization and Deaths Due to COVID-19

The fear of hospitalization and COVID-19 related deaths emerged as a significant motivator for adults in the family to get vaccinated against COVID-19 while not prioritizing childhood EPI vaccination. This fear was fueled by news reports highlighting the increasing trend of COVID-19 cases and related hospitalizations and fatalities. Within the community, this fear created a sense of urgency and a strong desire for protection against the virus.

During the COVID-19 pandemic, communities showed significant interest in the availability of vaccines, perceiving them as an important measure to protect against the virus. The widespread concern about infection and its potential health consequences may have influenced public health priorities. As a result, COVID-19 vaccination was often prioritized over routine childhood immunizations. This shift underscores the influence of perceived risk and urgency on vaccination behavior within affected populations.

“*I was in fear and thought that soon we all will be infected by the disease, and I shall die. I have quarantined myself for more than a month to avoid the disease. Every day there was news that our near relatives have been infected, and the other person died. It was a routine and every day the news of deaths was received*”.

(PRC–09, 59, M)

The local community witnessed a noticeable surge in the incidence of infections and subsequent hospitalizations. Nearly every household reported either confirmed or suspected cases of COVID-19. Although some individuals displayed symptoms, they received treatment from local healthcare practitioners within the community and did not seek care at hospitals.

During one of the interviews, a respondent was asked why they had not vaccinated their child, considering that all adult members of their family had received the COVID-19 vaccine. In response, the interviewee stated that,

“*Hospitals are full of COVID-19 patients and people are dying due to this disease, while I have never seen a child that has died due to not giving EPI vaccines. Therefore, everyone must get vaccines against COVID-19*”.

(PRC–14,24, M)

The surge in COVID-19-related fatalities served as a catalyst for heightened vaccination awareness within the community. In contrast, deaths attributed to vaccine-preventable diseases have not garnered similar attention, leading to a prevailing belief among community members that VPDs do not contribute significantly to hospitalizations and fatalities.

“*My child is almost more than 20 months old, and I have given only one dose of childhood vaccination at birth. The remaining doses were not given but the child is still alive and healthy, while I have seen many deaths in our colony due to COVID-19 in recent days*”.

(PRC–08,30, M)

Many members of the community held the opinion that if a child can lead a normal life without completing the full vaccination schedule, then the necessity of administering multiple doses to their children becomes questionable.

## 4. Discussion

Despite global efforts to promote immunization, little is known about the COVID-19 vaccination behavior of families whose children have defaulted from routine immunization under the EPI system. The findings of this study have the potential to offer novel insights into factors motivating vaccination compliance among families that would otherwise default from established vaccination programs. This study, conducted among 237 families of EPI defaulter children, showed that a very high proportion of family members within the households of EPI defaulter children had received COVID-19 vaccination, and >75% had received the complete 2-dose series. This finding is consistent with the overall COVID-19 vaccination coverage in Pakistan, which showed that 87% of 143 million individuals in the eligible population (≥12 years of age) had been fully or partially vaccinated against the disease in just over a year from the time COVID-19 vaccination activities began in Pakistan in February 2021 [17].

An analysis of characteristics of the defaulter children initially enrolled in the study found the highest frequency was for the second-born child, followed by first-born children, with a decline in the number of defaulters as birth order increased. Vaccination coverage is susceptible to influences stemming from child-related attributes, such as birth order, as well as other multifactorial elements such as social contextual factors and the nature of the healthcare system. A negative correlation between the child’s birth order and their vaccination status has been documented in other studies [18]. Additional reasons for low vaccination coverage in this population are discussed in a separate article, including the child’s illness at the scheduled time of vaccination, perceived side effects, caregiver-related factors, myths and misconceptions, vaccinator attitudes, overcrowded vaccination centers, and inadequate immunization service arrangements [16]. Although the proportion of family members who had received any COVID-19 vaccine was higher among males than females (87% among males versus 82% among females, *p* < 0.001), there was no significant difference by sex among those who had completed the two recommended doses of COVID-19 vaccine.

The qualitative assessment examined factors contributing to the discrepancy in vaccination rates between children who were not fully compliant with their EPI vaccination schedules and adults in the same family that received COVID-19 vaccines. Several key motivating factors were identified behind the willingness of adults to receive COVID-19 vaccines while not ensuring completion of the recommended EPI vaccination schedule for their children. Firstly, the imposition of mandatory COVID-19 vaccination for the eligible population. Although the EPI vaccination schedule for children is also mandatory, it lacks a robust mechanism for follow-up compliance like the system put in place for COVID-19 vaccination. Secondly, some family members admitted that they received COVID-19 vaccines without a full understanding of the vaccine’s benefits. Instead, their motivation for receiving the vaccines stemmed from the efficient follow-up mechanisms accompanying COVID-19 vaccination campaigns. These mechanisms linked essential services to COVID-19 vaccination, extending services to hospitals, bus terminals, airports, and other public places. Furthermore, the interviewees mentioned instances of individuals carrying COVID-19 vaccination cards as proof of vaccination, providing evidence when requested to verify their vaccination status. Interestingly, strict vaccination requirements within educational institutions, including schools, universities, and educational institutes, played a pivotal role in driving COVID-19 vaccination coverage. Additionally, government offices implemented entry-point checks of vaccination status, further promoting compliance. These findings are in line with a cross-sectional study in 2021 in Pakistan, which found that 48% of participants were willing to get vaccinated if mandated by the government [19]. Another study found that implementation of mandatory policies in several European countries had a positive impact on vaccination compliance and the potential to significantly reduce the prevalence and incidence of VPDs and their associated outbreaks in countries where standard vaccination policies had been ineffective to enhance childhood vaccination rates [20,21]. Thirteen European countries have mandatory vaccination policies for at least one antigen. Ten countries mandate a broad set of vaccines including diphtheria, tetanus, pertussis, hepatitis B, Hemophilus influenzae type b (Hib), poliomyelitis, measles, mumps and rubella [22]. In Australia, mandatory vaccination policies have been enacted to bolster vaccination coverage, primarily through school immunization laws [23]. Australia introduced the “No jab—No pay” initiative, wherein family tax benefits were linked to vaccination status, resulting in a notable increase in vaccination coverage [24].

Another prominent theme identified in our study centered on the implementation of house-to-house vaccination campaigns against COVID-19. This inclusive approach targeted both male and female members of the household, with the establishment of fixed vaccination centers being a precursor to the formation of mobile vaccination teams. These teams embarked on door-to-door visits to administer vaccines to family members who had not yet been vaccinated. Notably, this strategy proved to be a crucial factor in motivating eligible family members to receive COVID-19 vaccinations. While there are periodic outreach sessions for EPI vaccination of eligible and defaulter children, these sessions are usually confined to predetermined locations within the community [16]. Vaccinators would visit the designated village or colony and identify a central point where eligible or defaulter children would congregate for vaccination. Study participants highlighted that the key differentiator was the door-to-door approach for COVID-19 vaccinations. A study conducted in Kenya showed that coverage of nearly all EPI antigens experienced significant improvements following the implementation of door-to-door vaccination drives. Specifically, the study reported the following improvements: BCG vaccine coverage increased from 47.4% to 89.9%, Penta-1 vaccine coverage improved from 96.2% to 102%, Penta-3 vaccine coverage surged from 92.3% to 112.1%, and Measles-Rubella vaccine coverage rose from 81.7% to 111.5% [25].

Another theme that emerged in our study was that the community exhibited heightened perception of risk with COVID-19 vaccination when compared to childhood EPI vaccination. The same was seen in another study that fear of the disease was one of the reasons for uptake of typhoid vaccines [26]. During the peak of the COVID-19 pandemic nearly all elective health services, including routine outpatient department visits, were suspended. Human resources and healthcare facilities were redirected exclusively to the treatment and management of COVID-19 patients, leaving only emergency services available for non-COVID-19 patients. This shift in healthcare priorities induced a palpable sense of fear within the community regarding COVID-19 [27]. The release of daily situation reports from local hospitals, district, provincial, and national authorities, detailing COVID-19 case counts and mortality statistics, further exacerbated anxiety and fear within the community. These factors collectively acted as powerful motivators for the community to actively seek COVID-19 vaccination. During uncertain circumstances and when confronted with genuine threats, it is a natural human response to experience heightened levels of fear, anxiety, and stress. Understandably, these emotional responses intensified within the community during the COVID-19 pandemic, driven by the surge of COVID-19 infections, increased hospitalizations, and fatalities [28,29]. In the context of COVID-19 vaccination coverage, this heightened risk perception had a positive influence. A substantial portion of the community sought COVID-19 vaccination due to these fears, resulting in increased vaccination rates against the disease. Participants in our study, however, did not share the same heightened risk perception and anxiety about other vaccine-preventable diseases covered under the EPI childhood immunization schedule.

The findings of our study have important implications for policy and future research. Strengthening follow-up mechanisms, incorporating community-based outreach strategies, and exploring the ethical application of mandatory vaccination policies could enhance childhood immunization coverage in defaulter populations. Future research could examine the role of risk perception in vaccine acceptance and interventions to address barriers to routine immunization, particularly in settings with high adult vaccination coverage but low childhood vaccine uptake.

This study has several limitations. The study was conducted among participants from a single district in a Pakistan province with low routine immunization coverage and high prevalence of VPD outbreaks, which limits the generalizability of the findings to the entire population of Pakistan. Additionally, the COVID-19 vaccination status of the family members of defaulter children was self-reported, which may introduce recall and social desirability biases. Third, the respondents to the first component of our study were predominantly male, with only 20% (n = 53) representing females, and the male predominance was even higher for the qualitative study (only 2/16 interviewees were female). This skewed gender distribution was probably not significant in its impact on the information on vaccination status and demographic characteristics, as respondents relied on information provided by other family members, within their households, to corroborate such information. However, it may have introduced a bias in the selection and prioritization of reasons given for receiving COVID-19 vaccination in the qualitative assessment. Despite these limitations, we are confident that the findings of our study contribute to an understanding of attitudes towards COVID-19 vaccination among EPI defaulter families in Pakistan and similar contexts, since it is plausible that similar factors would inform vaccination compliance in other settings.

## 5. Conclusions

Our study revealed substantial disparities in COVID-19 and routine vaccination coverage within the same households of families with EPI defaulter children. Quantitative findings indicated that child-related factors, such as age, birth order, and number of children in the household, were associated with incomplete EPI vaccination, while household-level COVID-19 vaccination coverage was comparatively high. Qualitative findings help explain these patterns: mandatory COVID-19 vaccination policies, rigorous follow-up mechanisms, door-to-door campaigns, and heightened risk perception drove adults to vaccinate themselves, even when childhood vaccination remained incomplete. The integration of quantitative and qualitative evidence suggests that structural and behavioral interventions—such as improved follow-up, mandatory policies, and community awareness campaigns—could bridge the gap in childhood immunization coverage. Ethical application of vaccination requirements, ease of access to vaccination services, and targeted community engagement to increase risk perception of vaccine-preventable diseases may enhance compliance with routine EPI schedules among defaulter families, ultimately improving vaccination rates.

## Figures and Tables

**Table 1:** Characteristics of Defaulter Children and Family Members.

Variable	Number	%
**Age of Child**		
Below 6 months	28	11.8
6–12 months	140	59.1
13 to 18 months	49	20.7
Above 18 months	20	8.40
**Number of total children below 2 years in the household**		
1 child	139	58.6
2 children	68	28.7
3 or more children	30	12.7
**Gender of Child**		
Male	124	52.3
Female	113	47.7
**Residence**		
Urban	127	53.6
Rural	110	46.4
**Family type**		
Joint	208	87.8
Nuclear	29	12.2
**All male family members have completed two or more COVID-19 vaccine doses**		
Yes	129	54.4
No	108	45.6
**All female family members have completed two or more COVID-19 vaccine doses**		
Yes	160	67.5
No	77	32.5

**Table 2:** Comparison of Child and Household Characteristics by Routine Immunization Status and COVID-19 Vaccination Coverage Among Family Members.

Variables	None of the Children Is Fully Vaccinated		One or More Children Are Fully Vaccinated		
	Number	%	Number	%	*p* Value
**Age of Child**					
Below 6 months	17	10.5	11	14.7	0.047
6–12 months	93	57.4	47	62.7	
13–18 months	41	25.3	8	10.7	
Above 18	11	6.8	9	12	
**Number of total children below 2 years in the household**					
1 child	138	85.2	1	1.3	<0.001
2 children	19	11.7	49	65.3	
3 or more children	5	3.1	25	33.3	
**Gender of Child**					
Male	86	53.1	38	50.7	0.729
Female	76	46.9	37	49.3	
**Residence**					
Urban	88	54.3	39	52	0.739
Rural	74	45.7	36	48	
**Family type**					
Joint	149	92	59	78.7	
Nuclear	13	8	16	21.3	0.004
**All male family members have received two or more COVID-19 vaccine doses.**					
Yes	80	49.4	49	65.3	0.022
No	162	50.6	26	34.7	
**All female family members have received two or more COVID-19 vaccine doses.**					
Yes	108	66.7	52	69.3	0.684
No	54	33.3	23	30.7	

**Table 3:** Association of Child’s Age and Family Type with Routine Immunization Completion: Bivariate and Multivariate Analysis.

Variables	Unadjusted OR (95% CI)	*p*-Value	Adjusted OR (95% CI)	*p*-Value
Age of Child				
Below 6 months	1 (ref)	--	1 (ref)	--
6–12 months	0.78 (0.34–1.80)	0.562	0.81 (0.35–1.91)	0.637
13–18 months	0.30 (0.10–0.88)	0.028	0.34 (0.11–1.01)	0.052
Above 18 months	1.26 (0.40–4.04)	0.692	1.15 (0.35–3.80)	0.822
Family Type				
Joint	0.32 (0.15–0.71)	0.005	0.36 (0.16–0.81)	0.014
Nuclear	1 (ref)	--	1 (ref)	--
All male family members received ≥2 COVID-19 vaccine doses				
Yes	1 (ref)	--	1 (ref)	--
No	0.52 (0.29–0.91)	0.023	0.58 (0.32–1.03)	0.065

OR = Odds Ratio; CI = Confidence Interval; ref = Reference category.

## Data Availability

The original contributions presented in this study are included in the article. Further inquiries can be directed to the corresponding authors.
